# Synthetic biology enables mushrooms to meet emerging sustainable challenges

**DOI:** 10.3389/fmicb.2024.1337398

**Published:** 2024-02-13

**Authors:** Gen Zou, Tian Li, Ivan Mijakovic, Yongjun Wei

**Affiliations:** ^1^Institute of Edible Fungi, Shanghai Academy of Agricultural Sciences, Southern Key Laboratory of Edible Fungus Resource Utilization, Ministry of Agriculture, National Engineering Research Center of Edible Fungi, Shanghai, China; ^2^School of Pharmaceutical Sciences, Laboratory of Synthetic Biology, Food Laboratory of Zhongyuan, Zhengzhou University, Zhengzhou, China; ^3^Division of Systems and Synthetic Biology, Department of Biology and Biological Engineering, Chalmers University of Technology, Gothenburg, Sweden; ^4^Novo Nordisk Foundation Center for Biosustainability, Technical University of Denmark, Lyngby, Denmark

**Keywords:** mushroom, synthetic biology, sustainable development, circular economy, vegan meat, vegan leather

## Abstract

With the increasing sustainability challenges, synthetic biology is offering new possibilities for addressing the emerging problems through the cultivation and fermentation of mushrooms. In this perspective, we aim to provide an overview on the research and applications mushroom synthetic biology, emphasizing the need for increased attention and inclusion of this rapidly advancing field in future mushroom technology over China and other countries. By leveraging synthetic biology, mushrooms are expected to play a more versatile role in various area, including traditional fields like circular economy, human wellness and pharmaceutics, as well as emerging fields like vegan meat, mushroom-based materials and pollution abatement. We are confident that these efforts using synthetic biology strategies have the potential to strengthen our capacity to effectively address sustainable challenges, leading to the development of a more sustainable social economy and ecology.

## 1 Background

China’s Ministry of Science and Technology recently released new guidelines for the National Science and Technology Major Project of China which includes seven unprecedented projects related to mushroom-forming fungi in 2023. These projects highlight the growing interest of the government in the application of edible fungi and are likely to attract capital investments in the near future. They target the directional breeding of mushrooms using advanced synthetic biology techniques, the production of alternative meat based on edible fungi through advanced bio-manufacturing, and other mushroom-related initiatives. Herein, we outline the sustainable challenges associated with these projects and provide insights into the emerging synthetic biology paradigm for the future exploitation of mushrooms.

## 2 Circular economy

Owing to climate change and the high competitiveness of food marketing, the agri-food industry faces multiple challenges. Hence, the search for economically sustainable solutions is of great interest. These challenges particularly affect small-to-medium-sized farms, which face challenges in selling products and competing with large agricultural enterprises, since the efficiency of the current food production cycles in rural areas is relatively low. Circular economy provides a reliable solution to make the “take-make-waste” system more sustainable, eco-friendly, and provident, which involves the extraction of raw materials, the manufacturing of goods, and the disposal of by-products and end products through landfills, incinerators, and bodies of water worldwide. According to the reports, the future of agriculture will be characterized by the adoption of sustainable practices such as implementing a closed loop system, minimizing by-products and waste, and promoting eco-friendly production processes (Rahmann et al., 2021). These advancements are expected to support the survival of local agri-businesses and contribute to environmental protection (Stahel, 2016). Utilizing by-products and waste as a new source for food conversion is a promising practice due to their high nutritional content. This approach can contribute to the development of a circular economy and help to ensure food security (Zaini et al., 2023). Recently, production processes have been optimized from a circular economic perspective.

**Figure 1:**
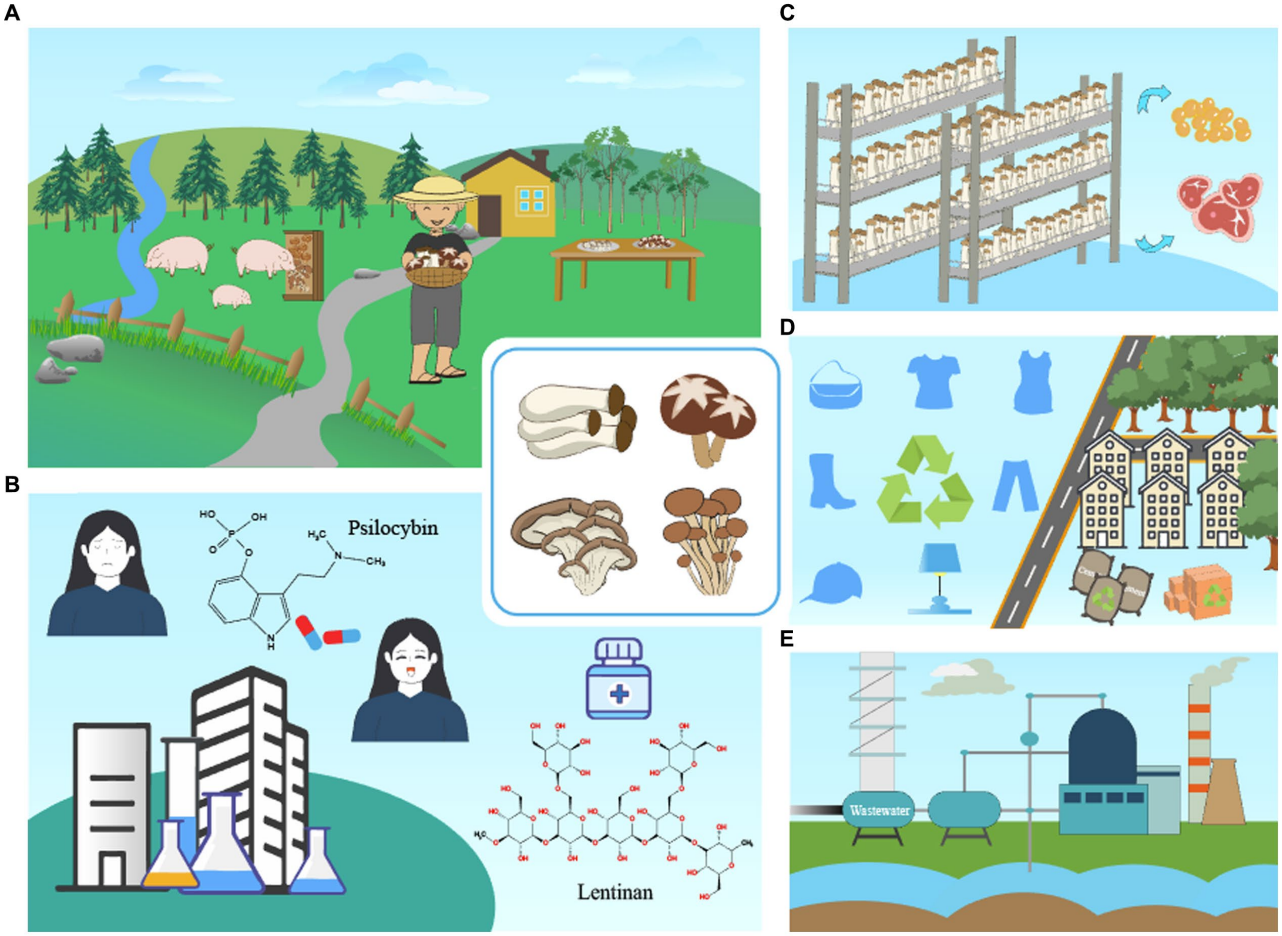
Illustration of synthetic biology enables mushrooms to meet emerging sustainable challenges. By harnessing the power of synthetic biology, mushrooms offer innovative solutions that address numerous sustainable challenges. These include circular economy **(A)**, human wellness and pharmaceutics **(B)**, vegan meat **(C)**, mushroom-based materials **(D)**, and pollution abatement **(E)**, which provide opportunities to build a more sustainable future.

Humans have collected and cultivated mushroom-forming fungi for millennia, primarily for food and medicine. Currently, most commercial mushrooms are saprotrophic fungi that grow on decaying organic matter, with wood being the most commonly utilized substrate. In China, mushroom cultivation, particularly that of *Lentinula edodes*, commonly known as xianggu or shiitake, has evolved to incorporate a substitute cultivation technique as an alternative to the traditional log cultivation. Agro-residues, such as sawdust, cottonseed hulls, and straw, are frequently utilized as suitable mushroom cultivation substitutes for wood (Li and Xu, 2022). Consequently, the emerging sustainable circular agricultural economy centered on mushrooms has shown promising progress (Figure 1A). Nevertheless, certain limitations remain when fully replacing the traditional agricultural economic model: (i) the main cultivated mushrooms (*L. edodes*, *Agaricus bisporus*, *Flammulina filiformis*) take months to grow and require specific conditions, such as low temperature, humidity, light, or other physical stimulation for fruiting; thus, mushroom cultivation is a slow, energy-consuming, and labor-intensive process; (ii) the conversion efficiency of substrates is low; and (iii) strategy is lacking for spent mushroom substrates (SMS) recycling (Zou et al., 2023). Traditional biotechnology alone is insufficient to address the challenges presented in situations such as mushroom cultivation in China, where the factories have become high-carbon enterprises due to factors such as poor temperature control. As a result, the resulting SMS is often not efficiently treated, leading to water and soil pollution, e.g., microplastic release, and water eutrophication (Marín-Benito et al., 2016). Currently, genome editing techniques are used in the precision molecular breeding of mushrooms. This approach holds great promise for customizing mushroom strains with enhanced substrate conversion rates and improved environmental adaptability, thereby facilitating the advancement of circular agricultural economic practices (Dong et al., 2022).

## 3 Human wellness and pharmaceutics

Mushrooms are a valuable source of proteins, fibers, lipids, minerals, and vitamins that are essential for a healthy human diet. Furthermore, these fungi can synthesize significant amounts of bioactive metabolites and components that play vital roles in human nutrition and can contribute to the prevention of various diseases. For centuries, mushrooms have been consumed by humans for cultural, medicinal, recreational, and religious purposes, extending beyond their use as food. The ancient Greeks strongly believed that mushrooms possessed the power to enhance the strength of soldiers during times of war (Valverde et al., 2015). The ancient Romans further elevated the status of mushrooms, considering them as the “food of the gods” and reserving their consumption for special festivals (Ahmed et al., 2023). In East Asian civilizations, mushrooms have been revered as a nourishing food for centuries and are referred to as the “elixir of life” (Mortimer et al., 2012). The Aztecs regarded hallucinogenic mushrooms (mainly *Psilocybe cubensis*) as teonanacatl, meaning “god’s flesh,” and utilized them in religious and healing ceremonies (Singh et al., 2022).

There is growing interest in exploring the potential health benefits of mushrooms for anti-cancer strategies and reducing the incidence of cardiovascular disease. This is primarily attributed to the immune priming or modulatory properties of β-glucans found in mushrooms. The clinical efficacy of β-glucan has been approved as an adjuvant therapy for cancer treatment (Zhang et al., 2019). Potential therapeutic properties, including obesity and diet regulation, gastrointestinal effects, cardiovascular and diabetes risk reduction, and epidermal damage repair are of concern. The US Food and Drug Administration (FDA) has approved β-glucan as a cholesterol-reducing food (Othman et al., 2011). However, mushroom β-glucans exhibit high variability in composition and structure, which likely affects the consistency of their therapeutic effects.

Psilocybin, a natural tryptamine compound found in psychedelic mushrooms (*Psilocybe*, *Conocybe*/*Pholiotina*, *Gymnopilus*, *Panaeolus*, etc.) (Bradshaw et al., 2024), shares a similar structure and mode of action as serotonin. Despite being a Schedule I drug in the US, there is renewed interest in its therapeutic use owing to its relatively low harm profile compared to similar substances such as ketamine. The FDA has designated psilocybin as a breakthrough therapeutic agent for the treatment of major depressive disorders (Reiff et al., 2020). Due to historical, legal, and cultural differences, psilocybin is still classified as a harmful substance in most countries.

Furthermore, mushrooms generate numerous natural compounds with great potential as lead compounds in the development of medicines (Figure 1B). These include lovastatin, ergosterol, cordycepin, pentostatin, chitosan, eritadenine, indoles, tocopherols, γ-aminobutyric acid, and ergothioneine. Moreover, some of them have shown valuable functional activity and can be efficiently obtained from mushrooms. Despite the enormous potential of medicinal mushrooms for producing bioactive substances, limitations in classic biotechnology, such as large fragments of genes, cryptic clusters, and lacking efficient genetic manipulation in mushrooms, have prevented the full elucidation and characterization of their synthetic pathways. As a result, most medicinal mushrooms are still used as traditional herbs, which suffer from well-known limitations such as unknown side effects and unclear pharmacokinetics.

With the advancements in synthetic biology, mushroom-based cell factories, which can effectively and sustainably transform agricultural waste into a wide range of diversified products, are expected to be developed (Zou et al., 2021). Ergothioneine, an unusual thio-histidine betaine amino acid with potent antioxidant activity, has already been produced in mushroom cell factories as a paradigm, demonstrating the promise of mushroom-based cell factories to produce valuable natural products (Cheah and Halliwell, 2021).

## 4 Vegan meat

Currently, millions of families worldwide cannot obtain sufficient food because of geopolitical conflicts, population growth, and climate extremes. The agricultural sector requires innovation and reform to meet the growing demand for food. The expansion of agri-food production has caused significant harm to the environment and contributed 34% of the anthropogenic greenhouse gas emissions (GGE) (Crippa et al., 2021). This has led to substantial changes in biodiversity and climate change (Pearman et al., 2024).

The nutritional value of fungal biomass is being increasingly recognized owing to its low levels of fat, salt, cholesterol, and calories. There are abundant bioactive compounds and essential nutrients in mushrooms, such as fibers, proteins, minerals, vitamins, and nutraceuticals, making fungal biomass a suitable dietary option for human consumption (Figure 1C). Edible fungi can be cultivated using sustainable substrates, such as agricultural residues and their by-products, leading to a balanced and nutritious food system while reducing the adverse effects associated with traditional food production, including soil pollution, water pollution, and greenhouse gas release. Moreover, the organoleptic properties of mushroom biomass, including flavor and texture, make it a particularly well-suited meat alternative to mycelial biomass derived from ascomycetes since 1960s (Nordling, 2022).

Despite being developed for over half a century, mycoprotein has not gained widespread consumer acceptance primarily due to its flavor (Finnigan et al., 2019). Most fungi have a characteristic mushroom smell or a musty odor caused by mushroom alcohol (1-octen-3-ol), but they lack the “meaty” flavor associated with heme-iron. Therefore, future mushroom-based vegan meats should explore the use of synthetic biology to enable fungi to be customized, creating new “fungal species” with a flavor more similar to that of meat. On the other hand, certain mushrooms (e.g., *Boletus edulis*, *Amanita caesarea*, *Tuber melanosporum*) are charming in gastronomy due to their distinct flavor and aroma. Enhancing these prized characteristics, as well as nutritional content, through the application of synthetic biology would be of great significance.

## 5 Mushroom-based materials

With the evolution of social norms and the development of environmental sustainability, leather, which is often considered a byproduct of meat production, has drawn criticism for its social contradictions and environmental drawbacks (Tewari et al., 2023). Livestock breeding is associated with grazing deforestation, which contributes to considerable GGE and environmental damage caused by animal waste. Leather processing uses various hazardous chemicals to generate substantial quantities of sludge waste. These issues have prompted the development of nonanimal leather-like materials. Mushroom-based biomass is an emerging solution that shows great promise as a cost-effective, socially, and environmentally responsible alternative to bovine leather and synthetic chemical leather (Jones et al., 2020). Mushroom-based leather alternative can be used in various fields such as automotive interiors, apparel, bags, and suitcases. Leather-like materials derived from mushrooms exhibit more comparable appearance and material properties to those of bovine leather than other synthetic leathers (Tewari et al., 2023).

Mushrooms are being explored for other innovative applications such as mycelium-based composites that can be used in building materials or packaging (Figure 1D) (Alemu et al., 2022). The mycelium serves as a natural adhesive and is formed by individual hyphae that develop from the spores of the mycelial fungal strain. The creation of mycelium-based composites involves several steps. First, the substrate is homogenized to enhance the growth surface area, and then sterilized to eliminate microbial contamination. Next, a controlled environment is created for fungal colonization using an inoculation procedure, followed by dehydration and characterization.

New mushroom-based alternative materials primarily consist of chitin and other polysaccharides, including glucans, proteins, chitosan, polyglucuronic acid, and lignocellulose. One of the key advantages of these materials is that they are fully biodegradable (Jones et al., 2020). However, the main challenge in advancing and developing mushroom-based materials is obtaining better shine and toughness of leather. Tallow oil, an essential ingredient in maintaining luster and preventing dry cracking, is lacking in fungal mycelium. Therefore, customizing the composition of mycelium leather through synthetic biology is the main direction for optimizing products in the future. As an example, one could introduce oil synthetic pathway to enhance the luster of mycelium leather, while also modulating the mycelial protein content to prevent mildew.

## 6 Pollution abatement

Mushrooms are known for their ability to produce considerable amounts of non-specific oxidative lignocellulolytic enzymes (Gong et al., 2023). Mycelia are porous materials with excellent adsorption capacities. Consequently, mushrooms efficiently degrade and remove complex pollutants, including pesticides, pharmaceutical compounds, heavy metal ions, and microplastics (Figure 1E). Although bacterial processes currently dominate pollution abatement, the exploratory use of mushrooms for pollution treatment has produced favorable outcomes, surpassing those of bacteria-based methods. The versatility and non-specific enzymatic cocktail (mainly the oxidative species of CAZy) of mushrooms enable them to degrade a wide range of contaminants, giving them an advantage over bacteria-based treatments. Certain antibiotic-rich (ofloxacin, ciprofloxacin, etc.) pollutants may render bacterial treatment ineffective; however, mushrooms are not affected by most commercially available antibiotics (Benjamin et al., 2017). Inactivated (dead or dormant) mushroom mycelia or other tissues exhibit excellent adsorption of heavy metals and chemicals because of the long-lasting polyporous structure of fungal biomass (Mir-Tutusaus et al., 2018). Furthermore, this process does not require additional matter or energy to support growth.

Despite their high performance in pollution removal, mushroom-based treatments have a significant drawback in that they require a long retention time, typically 7–14 days (Křesinová et al., 2018). In contrast, the traditional bacteria-based treatment processes typically require a few hours. This prolonged retention time poses a challenge when incorporating mushroom treatments into existing pollution abatement strategies (Nguyen et al., 2013). Another hurdle in ensuring the reliability of mushroom treatment is the need for an acidic environment, preferably pH 4.5–7.0, which falls within the optimal range for mycelial growth (Huang, 2019). Addressing these challenges is crucial for the effective implementation of mushroom-based treatment methods for pollutants in the near future.

## 7 Conclusion

With the continuous advancement of biotechnology and our deepening understanding of mushrooms, we are entering an exciting era of mushroom synthetic biology. In this perspective, we aim to shed light on this emerging paradigm, which hold great promise for harnessing mushrooms in various domains. Furthermore, a brief outline of potential areas for further research in these domains is provided. It is our hope that mushroom synthetic biology will significantly contribute to creating a better and more sustainable world. To achieve this goal, it is imperative to prioritize the development of additional mushroom synthetic biology tools and explore diverse applications across different fields. Taken together, these efforts have the potential to accelerate our ability to address sustainable challenges effectively.

## Data availability statement

The original contributions presented in the study are included in the article/supplementary material, further inquiries can be directed to the corresponding author.

## Author contributions

GZ: Conceptualization, Funding acquisition, Project administration, Writing – original draft. TL: Investigation, Writing – review & editing. IM: Funding acquisition, Writing – review & editing. YW: Conceptualization, Funding acquisition, Project administration, Validation, Writing – original draft, Writing – review & editing.
